# Challenges in Diagnosis and Therapeutic Strategies in Late-Onset Multiple Sclerosis

**DOI:** 10.3390/jpm14040400

**Published:** 2024-04-10

**Authors:** Viviana Nociti, Marina Romozzi, Massimiliano Mirabella

**Affiliations:** 1 Centro Sclerosi Multipla, Fondazione Policlinico Universitario Agostino Gemelli IRCCS, 00168 Rome, Italy; massimiliano.mirabella@policlinicogemelli.it; 2 Dipartimento Universitario di Neuroscienze, Università Cattolica del Sacro Cuore, 20123 Rome, Italy; marinaromozzi@gmail.com

**Keywords:** late-onset multiple sclerosis, personalized medicine, disease-modifying therapies, comorbidities

## Abstract

Multiple sclerosis (MS) is a chronic inflammatory and degenerative demyelinating disease of the central nervous system of unknown etiology, which affects individuals in their early adulthood. However, nearly 5–10% of people with MS can be diagnosed at ages above 50 years old, referred to as late-onset multiple sclerosis (LOMS). Some studies have reported a distinctive presentation, clinical course, and prognosis for LOMS, implicating a different diagnostic and therapeutic approach for this population. Furthermore, similar manifestations between LOMS and other age-related conditions may lead to potential misdiagnosis and diagnostic delays, and a higher burden of multimorbidity associated with aging can further complicate the clinical picture. This review aims to explore the clinical characteristics, the disease course, and the differential diagnosis of LOMS and addresses therapeutic considerations for this population.

## 1. Introduction

Multiple sclerosis (MS) is a chronic inflammatory and degenerative demyelinating disease of the central nervous system (CNS) of unknown etiology [1]. The pathophysiology of MS is characterized by an altered multidirectional interaction among different immune cell types in the periphery and resident CNS cells [2,3]. It is most commonly diagnosed between the ages of 20 and 40 [1]. However, in approximately 3–5% of the cases, MS can have a pediatric onset, and nearly 5–10% of people with MS (PwMS) can be diagnosed at ages above 50 years old, referred to as late-onset multiple sclerosis (LOMS) [4,5]. When the presentation occurs after the age of 60, it is defined as very late-onset MS [6].

Several studies have shown that LOMS has been increasingly diagnosed in recent years, contributing to its better recognition and characterization [7,8]. However, similar manifestations between LOMS and other age-related conditions may lead to potential misdiagnosis and diagnostic delays [9]. Furthermore, a higher burden of multimorbidity associated with aging can further complicate the clinical picture [10,11].

Besides genetic susceptibility, several environmental risk factors have been associated with an increased risk of MS, such as Epstein–Barr virus infection, smoking, obesity during adolescence, and vitamin D deficiency [12,13]. In this context, the increasing prevalence of LOMS may be explained by a complex interplay between several environmental factors and lifestyle changes over time (e.g., increasing smoking habits and obesity prevalence and infections) [8]. Another contributing factor could be the increased accessibility of brain magnetic resonance imaging (MRI) and updated diagnostic criteria based on MRI, facilitating a more precise differentiation between demyelinating lesions and vascular or nonspecific ones [8].

Some studies have reported a distinctive presentation, clinical course, and prognosis for LOMS, which implicates a different diagnostic and therapeutic approach for this population [14].

The treatment of LOMS poses unique challenges. Although current disease-modifying therapies (DMTs) for MS effectively reduce neuroinflammation, they may not be as effective in reducing the disability accumulation associated with neurodegeneration, which is more prevalent in older individuals with MS [15].

Clinical trials for the currently approved DMTs have excluded individuals over 50–55 years with MS, and the only data available are retrievable from subgroup analyses [16].

Despite these limitations, DMTs are frequently prescribed for LOMS in clinical practice, although evidence regarding their safety and efficacy in this population is scarce and primarily derived from real-world observational studies [17,18]. 

This review aims to explore the clinical characteristics, the disease course, and the differential diagnosis of LOMS, and addresses therapeutic considerations for this population. 

## 2. Methods

To conduct this narrative review, a broad literature search was conducted across multiple databases to identify relevant studies on MS and LOMS published up to February 2024. In addition, we manually checked the bibliographies of relevant papers and reviews to locate any other pertinent articles. Finally, the extracted data were organized into spreadsheets.

## 3. Clinical and Radiological Characteristics and Disease Course of LOMS

Late-onset MS is considered a rare phenomenon; however, the reported prevalence among PwMS ranges between 4 and 10% in different studies, with LOMS appearing more prevalent in females but with an attenuated female/male ratio compared to adult-onset MS [19,20]. The initial presentation is more often monosymptomatic with motor deficits, with less than 20% of LOMS manifesting visual system involvement as the first manifestation [6,21,22]. As the disease progresses in individuals with LOMS, there is a progressive involvement of other functional systems, particularly with an increase in sensory and sphincteric disturbances [23].

The primary progressive course is the most frequent disease phenotype, while in up to 40% of the cases the presentation is relapsing–remitting [5,24,25]. However, patients presenting an acute clinical attack usually have a short interval to the beginning of a progressive phase or a second attack [22]. The mean duration of the relapsing–remitting period and the mean time from the onset of the disease to the start of the secondary progressive phase tend to be shorter in LOMS patients compared to those with adult-onset MS [26]. Additionally, individuals with LOMS experience fewer relapses within the first two years following diagnosis compared to adult-onset MS patients [26].

While some studies suggest a poorer prognosis for LOMS, others indicate no significant difference between LOMS and adult-onset MS [14,27,28]. At the time of diagnosis, individuals with LOMS typically present with significantly higher EDSS scores compared to those with adult-onset disease [26]. Furthermore, most studies describe a rapid progression of the disability in this population, with a higher rate of reaching the EDSS milestone 6.0 [29]. Some studies suggest that sex is not associated with different trajectories of progression in LOMS [24].

In a study by Tremlett and Devonshire, people with LOMS took a median of 16.9 years to reach an EDSS score of 6.0, whereas those with adult-onset MS required 27.7 years [27]. However, people with LOMS were older at the time of reaching an EDSS score of 6.0 (71.2 years old compared to 58.4 years old in adult-onset MS) [27]. Furthermore, deterioration due to normal aging in this population could have acted as a confounding factor accelerating disease progression.

Regarding brain MRI findings, spinal lesions are more frequently found in the LOMS group, with transverse myelitis being a common presentation [25]. Studies including individuals with LOMS reported spinal lesion rates ranging between 35% and 80% [25,26]. Conversely, infratentorial lesions are significantly more prevalent in PwMS with an earlier onset compared to LOMS [25]. In individuals with LOMS, spinal lesions and spinal gadolinium-enhancing lesions appear to be predictors of disability progression [26]. However, the percentage of individuals with a gadolinium-enhancing lesion at the diagnosis slightly decreases with advancing age, as contrast enhancement is related to blood–brain barrier disruption and the active inflammatory phase of the disease, implying a potential decline of inflammatory processes with age [26]. 

Additionally, some authors have attributed the higher prevalence of primary progressive onset in LOMS to the existence of a latent period, during which the inflammatory relapsing–remitting phase either remains clinically silent or does not occur before the apparent presentation of primary progressive MS [27]. Furthermore, accumulating evidence suggests that the clinical course of MS is better considered as a continuum with concurrent pathophysiological processes variably contributing across individuals and over time [30].

An aging brain and immune system with altered T-cell and B-cell activity and impaired oligodendrocyte progenitor recruitment and differentiation might also lead to inadequate remyelination and the incapability to manifest the characteristic relapsing–remitting phase [31]. The decline in immune system function linked with natural aging, known as immunosenescence, may contribute, at least in part, to the shift in the disease progression from an inflammatory to a neurodegenerative phenotype [18].

Some authors suggest that individuals diagnosed after the age of 50 may have experienced demyelinating events but did not seek medical attention, thus remaining undiagnosed until later [20]. Roohani et al. conducted a study in which they reclassified patients with LOMS who initially presented with primary progressive onset. Their reclassification revealed that a significant proportion of patients were inaccurately classified as having primary progressive MS instead of the secondary progressive form [20].

In terms of paraclinical findings, the proportion of LOMS patients with oligoclonal bands is relatively lower compared to those seen in juvenile-onset MS, where they are present in over 90% of the cases [21]. 

## 4. Comorbidities 

The aging process brings about various physiological changes that can predispose individuals to an increased risk of developing comorbid conditions. The risk of age-related multimorbidity considerably impacts patients over 50 years old and complicates the process of differential diagnosis [32,33,34]. 

It is noteworthy that MS is associated with a spectrum of multisystem comorbidities, regardless of the age of onset. These comorbidities encompass a range of conditions, including neurological disturbances, psychiatric disorders, cardiovascular diseases (e.g., hypertension, hyperlipidemia, cardiac arrhythmias, heart failure), autoimmune conditions, and metabolic disorders [32,33,34,35,36]. While many of these comorbidities are present either before or at the onset of MS symptoms, their prevalence appears to increase over time [37]. Several studies have shown that the presence of comorbidities can determine a delayed diagnosis and greater severity of the disability at MS diagnosis and an increased relapse rate during the disease course [38].

A cohort study involving 8983 PwMS enrolled in the North American Research Committee on Multiple Sclerosis Registry demonstrated that vascular comorbidities, whether present at symptom onset, diagnosis, or later in the disease course, were associated with an increased risk of disability progression in PwMS [39]. The presence of comorbid conditions amplifies the burden of disability experienced by PwMS, complicating treatment decisions and safety considerations and affecting overall quality of life and mortality rates [40,41,42]. 

Vascular comorbidities such as hypertension, type-2 diabetes, dyslipidemia, and cardiovascular disease have become increasingly prevalent in PwMS and can impact the clinical course of MS, potentially exerting an independent effect on disability accumulation [39]. Vascular risk factors have also been implicated in the etiology of cerebral small vessel disease characterized by typical MRI markers such as white matter hyperintensities and lacunes [43]. 

## 5. Differential Diagnosis

Several conditions have to be considered in the differential diagnosis of LOMS. With the large availability of brain MRI, the differential diagnosis is mostly made based on imaging findings. In a Spanish case series of eighteen people with LOMS, in six patients, mistaken diagnoses had previously been made of idiopathic trigeminal neuralgia, cervical myelopathy, and cerebrovascular disease [44]. 

### 5.1. Vascular Lesions

Certainly, the most common differential diagnoses of LOMS include vascular lesions, typically seen in patients of advanced age, such as lacunar infarcts or white matter hyperintensities of presumed vascular origin, that must be ruled out in case of coexistence of vascular and cardiac risk factors. In older MS patients, in fact, MRI specificity declines due to the co-occurrence of microangiopathic lesions. In the age group older than 50 years old, white matter signal changes occur in more than 50% of asymptomatic individuals and can mimic MS lesions on MRI [45,46]. One of the unique challenges faced among the aging MS population is the difficulty in differentiating mixed neuroimaging patterns with vascular and MS lesions, since both conditions can exhibit comparable radiological features [45]. White matter hyperintensities of presumed vascular origin are hyperintense in the T2 and fluid-attenuated inversion recovery (FLAIR) sequences; they typically occur bilaterally and symmetrically in the periventricular area, located adjacent to the lateral ventricular wall or in the deep white matter most frequently in the frontal and parietal lobes, basal ganglia, corona radiata, and centrum semiovale [47]. 

Lacunar infarcts are small-sized infarcts (3–20 mm) characterized by increased T2-weighted signal intensity, caused by occlusion of small deep penetrating branches of the large cerebral arteries, mostly occurring in the basal ganglia, thalamus, subcortical white matter, and pons [48]. Lacunar infarcts can be clinically silent or, less frequently, symptomatic [49]. The differentiating characteristics of MS and cerebral small vessel disease are reported in Table 1. 

In general, conventional MRI techniques are not able to certainly distinguish between demyelinating and ischemic lesions, but several radiological signs can help in the distinction from mimic diseases, including the presence of Dawson’s fingers, the central vein sign, and the coexistence of paramagnetic rim lesions. However, most of these radiological signs are better detectable using optimized susceptibility-weighted MRI sequences at high and ultra-high field strength that are still not regularly recommended for clinical use [50,51,52]. 

### 5.2. Primary and Secondary Central Nervous System Vasculitides

When an atypical MRI pattern is observed, primary angiitis of the central nervous system (CNS), also named primary CNS vasculitis, must be considered. Primary angiitis of the CNS is a rare inflammatory disorder of the blood vessels of the brain and the spinal cord. The second group of disorders that can mimic LOMS is secondary CNS vasculitis. This is a broad grouping that encompasses numerous infectious, rheumatic, and autoimmune disorders. A complete diagnostic workup for these secondary CNS vasculitides should also be conducted before confirming a diagnosis of LOMS [53].

The CNS involvement and related clinical features of vasculitides exhibit a considerable variability, ranging from focal neurological deficits to diffuse encephalopathy or involvement of multiple spinal segments [53].

Brain MRI usually shows multiple infarctions, typically bilateral, affecting different vascular territories with variable sizes. Hyperintense T2/FLAIR lesions involve both white and gray matter, primarily located in subcortical regions, many of which exhibit contrast enhancement. Hemorrhagic lesions, including subarachnoid hemorrhage, may also be present [54]. Digital subtraction angiography, computed tomography (CT) angiography, and MR angiography typically show focal or multifocal segmental narrowing and/or beading of both small and medium-sized blood vessels [53]. The presence of intense and simultaneous enhancement of the majority of brain MRI lesions during a clinically active phase of the disease is considered a crucial criterion for diagnosing vasculitis, although definitive diagnosis still relies on leptomeningeal–cortical brain biopsy. Some authors suggested that the central vein sign may also aid in differentiating inflammatory CNS vasculopathy from MS at standard clinical magnetic field strengths [55].

Another rare condition that may be considered in the differential diagnosis of LOMS is Susac syndrome, caused by immune-mediated occlusion of microvessels in the brain, retina, and inner ear, which are responsible for the characteristic clinical triad of CNS dysfunction, visual disturbances caused by branch retinal artery occlusions, and sensorineural hearing loss [56]. The typical age of onset of the disease ranges between 20 and 40 years of age. However, cases have been reported in individuals up to 70 years old [57].

Brain MRI plays a crucial role in distinguishing Susac syndrome from other conditions, including MS and acute disseminated encephalomyelitis (ADEM). The MRI abnormalities are mostly localized in the leptomeninges, grey matter, and white matter, particularly the corpus callosum, where lesions may become confluent and exhibit a classic “snowball” appearance [57].

### 5.3. Acute Disseminated Encephalomyelitis

Acute disseminated encephalomyelitis is an immune-mediated demyelinating disease of the CNS that typically follows a febrile infection or a vaccination, predominantly affecting children [58]. While ADEM is usually considered monophasic, recurrence with a multiphasic course occurs in 25–33% of patients [58]. Approximately half of the ADEM cases exhibit anti-myelin oligodendrocyte glycoprotein (MOG) immunoglobulin G antibodies, especially in cases with a multiphasic course [59,60]. 

Both ADEM and MS are disseminated CNS disorders, manifesting a broad spectrum of neurological signs. ADEM often presents with multiple symptoms and signs (polysymptomatic), whereas MS commonly presents with isolated symptoms [61,62]. Pyramidal, cerebellar, and brain stem signs are common in both disorders. Encephalopathy with depressed consciousness and altered sensorium are more common in ADEM (45–75%) than in MS (13–15%). Optic neuritis occurs in both ADEM and MS, typically bilateral in ADEM and unilateral in MS [63]. 

MRI is essential in differentiating between ADEM and MS. The lesions in ADEM are often asymmetric, poorly demarcated, and large (>1–2 cm), whereas MS lesions have well-defined margins. Periaqueductal, corpus callosum, and periventricular white matter lesions are characteristic of MS, while ADEM lesions tend to be located in the deeper white matter, with sparing of the periventricular regions. In addition, although the white matter is classically involved in both disorders, the grey matter (both cortical and deep grey/basal ganglia) is frequently involved in ADEM [62,64]. The MRI lesions in ADEM lesions usually exhibit contrast enhancement and restricted diffusion [62,64].

### 5.4. Myelopathies 

Cervical spondylotic myelopathy and related degenerative diseases of the spine are the most prevalent causes of spinal cord injury in older individuals [65]. Cervical spondylotic myelopathy is associated with a progressive deterioration of motor and sensory function. A differential diagnosis between inflammatory and compressive or vascular-related myelopathy due to spondylotic abnormalities should be considered in cases of progressive spastic paraparesis, at times asymmetric and often accompanied by paresthesia of the feet and/or hands, with or without urinary symptoms [65]. Differentiating MRI and laboratory features of myelopathy associated with MS and cervical spondylotic myelopathy are summarized in Table 2.

In the differential diagnosis of inflammatory myelopathies, several entities must be taken into account, including those associated with specific antibodies primarily targeting CNS antigens, such as anti-aquaporin-4 antibodies (AQP4), anti-MOG antibodies and the paraneoplastic myelopathies, and those associated with systemic autoimmune disorders having secondary CNS involvement (e.g., systemic lupus erythematosus, Sjogren’s syndrome, and sarcoidosis) [66]. In addition, immune-mediated myelopathies can occur after the administration of drugs affecting the immune system function (e.g., immune checkpoint inhibitors and TNF-alpha inhibitors) [67]. Specific radiological patterns to distinguish inflammatory myelopathies are summarized in a review by Cacciaguerra and colleagues [66].

Metabolic and toxic myelopathy should also be considered in older individuals. As a matter of fact, several nutritional deficiencies (cobalamin, folate, copper) and toxic substances (organophosphates, nitrous oxide, zinc) cause subacute-onset myelopathy while a chronic myelopathy may also result from vitamin E deficiency [68].

An infectious myelopathy in older individuals may arise from direct infection or parainfectious autoimmune-mediated mechanisms [69]. Several microorganisms are associated with myelopathy, including bacteria, viruses, fungi, and parasites. Epidemiologic risk factors, clinical characteristics, cerebrospinal fluid profiles, and imaging features may aid in the differential diagnosis of infectious myelopathies [69].

### 5.5. Optic Neuritis

Regarding optic neuritis presenting in older individuals, the differential diagnosis should include ischemic optic neuropathy, toxic and nutritional optic neuropathy, and infectious and autoimmune etiologies [70]. The autoimmune optic neuritis includes forms caused by AQP4 autoimmunity and anti-MOG-associated disease, characterized by bilateral optic neuritis, unlike MS [71]. Rare causes of autoimmune optic neuropathies, such as glial fibrillary acidic protein (GFAP) and collapsin response-mediator protein 5 (CRMP5) autoimmunity, should also be considered in patients presenting with bilateral painless optic neuropathy associated with optic disc edema [70,71].

### 5.6. Sarcoidosis

Neurologic involvement of sarcoidosis (neurosarcoidosis) includes a broad range of manifestations affecting optic nerves, spinal cord, hypothalamus, and single or multiple cranial nerves [72]. Brain parenchymal neurosarcoidosis may arise as a consequence of meningeal spread or vascular disease. Characteristic MRI findings include contrast-enhancing or T2 hyperintense and T1 isointense lesions, with or without contrast enhancement [72]. However, in neurosarcoidosis, leptomeningeal enhancement, parenchymal enhancing mass or nerve roots, and chiasmal enhancement are frequently observed [72].

## 6. Treatment Aspects 

### 6.1. Efficacy of DMTs

The aging of the MS population and the substantial proportion of patients presenting with LOMS have important clinical implications, particularly concerning treatment options [18]. Furthermore, the prevalence of progressive forms in LOMS poses challenges in terms of selecting appropriate treatments. Despite the expansion of the therapeutic options for MS, only a few DMTs are approved for treating progressive forms, with most demonstrating efficacy mainly in secondary progressive forms with inflammatory activity [73]. A meta-analysis has suggested that efficacy of DMTs on MS disability progression is strongly dependent on age. The model suggests that there is no predicted benefit from receiving immunomodulatory treatments after the age of 53 [74]. 

Individuals over 50–55 years old are often underrepresented in clinical trials, hence complicating the assessment of the risk–benefit profile of approved DMTs for this population [18]. Subgroup analyses based on the age of the comparative DMTs’ effectiveness were conducted in most phase 3 trials. Most DMTs show little to no effect on disability progression in patients older than 40 years compared to comparator arms [16]. While a few trials of first-line DMTs have shown a positive effect in older patients on markers of disease activity, such as the annualized relapse rate, the age cut-off was typically set at 40 years old, thus not fully representative of LOMS [75,76,77]. Regarding second-line treatments, in AFFIRM and SENTINEL trials, natalizumab failed to reduce progression in PwMS older than 40 years although it significantly reduced the annualized relapse ratio in patients older than 40 years compared to placebo [78].

Fingolimod and ozanimod demonstrated no significant reduction in disability progression or relapse rates in patients over 40 years old compared to those receiving a placebo [79].

Data on ocrelizumab on reducing the relapse rate and the disability progression are controversial in two clinical trials, OPERA and ORATORIO, with a notable trend to benefit younger subjects [80,81]. However, CONSONANCE, an ongoing open-label single-arm study evaluating the efficacy of ocrelizumab in patients with SPMS and PPMS, is the first trial that included patients up to 65 years old. 

A post hoc analysis from the randomized CARE-MS showed that alemtuzumab did not exhibit different efficacy in young and old patients in terms of relapse rate, disability, and MRI outcomes. However, age-related increases in serious infections, malignancies, and deaths were observed [82].

Siponimod, in a phase 3 clinical trial (EXPAND), included patients up to 60 years of age with SPMS and represented the first DMT that showed efficacy in reducing disability accumulation in SPMS [83]. 

Real-world studies on the topic are underrepresented. A retrospective study by Thakolwiboon and colleagues investigated the effectiveness and tolerability of DMTs in a cohort of 44 relapsing–remitting LOMS patients. The effectiveness was assessed by the no evidence of disease activity (NEDA) score after 12 months of therapy, and tolerability was reported as a discontinuation rate for safety alerts [84]. At 12 months, 68.4% of patients with glatiramer acetate remained in NEDA status as did 66.7%, 53.3%, 50.0%, and 45.5% of patients with dimethyl fumarate, teriflunomide, ocrelizumab, and interferons, respectively. During follow-up, 38.5% and 37% of patients taking glatiramer acetate and interferons were unable to tolerate the side effects, especially injection site reactions. Only 16.7%, 12.5%, and 5.3% of patients with dimethyl fumarate, ocrelizumab, and teriflunomide were switched therapy because of adverse events [84].

Zanghì and colleagues compared the effectiveness of injectable and oral first-line DMTs in relapsing–remitting LOMS and found no differences between the two investigated groups regarding the risk of relapse occurrence, disability progression, and treatment discontinuation [85].

In another study of relapsing LOMS, treatment with interferon beta did not reduce the progression of disability in this group of patients [86].

**Figure 1:**
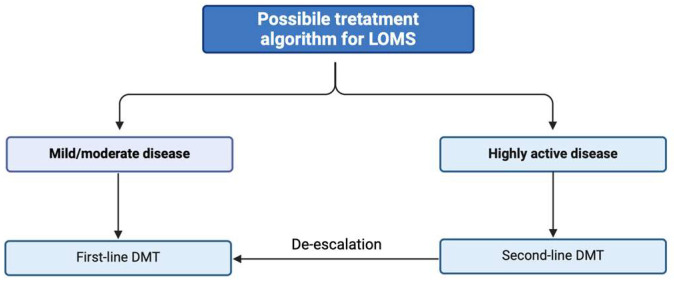
A possible algorithm of treatment strategies for late-onset multiple sclerosis (LOMS). Abbreviations: LOMS, late-onset multiple sclerosis; DMT, disease-modifying therapies.

**Figure 2:**
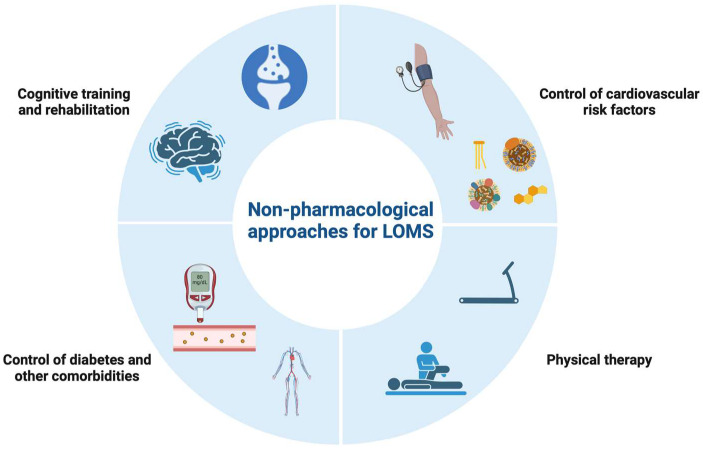
Possible non-pharmacological approaches for late-onset multiple sclerosis (LOMS). Abbreviations: LOMS, late-onset multiple sclerosis.

A possible algorithm of treatment strategies for LOMS is presented in Figure 1, along with potential non-pharmacological strategies (Figure 2).

### 6.2. Safety and Tolerability

The age-related changes that take place in the immune system (immunosenescence) generally result in a higher incidence of opportunistic infections and neoplasms. These changes can impact the safety profile of DMTs, resulting in a higher incidence of potentially severe adverse events among elderly patients [18]. 

Advanced age is an independent risk factor for the development of progressive multifocal leukoencephalopathy (PML) [87,88]. Elderly individuals are more susceptible to PML following fewer natalizumab infusions and exhibit higher mortality rates. Prosperini et al. investigated if age at treatment start affects the time to onset of natalizumab-related PML. The authors showed that patients older than 50 years had a more than doubly increased risk for an earlier PML onset [88].

Infections and the reactivation of latent viruses are more common among older individuals, both in the general population and in PwMS. Specifically, the risk of varicella-zoster virus infection associated with sphingosine-1-phosphate receptor modulators, cladribine, and alemtuzumab increases with age [89].

A recent meta-regression also demonstrated a higher neoplasm rate in patients over 45 years of age with depletive agents (alemtuzumab, cladribine, ocrelizumab) [90]. 

Additionally, the accumulation of comorbidities associated with aging can further influence the safety of DMTs [18]. For example, hypertension is a potential adverse event of teriflunomide, fingolimod, and ozanimod, and is also more frequent with aging [91]. The negative chronotropic effects of fingolimod might also be age-dependent [92]. 

Since there is little information on the efficacy of DMTs in older people, elderly PwMS should be treated following the same treatment algorithms used in younger people. Regardless of age, when inflammatory activity is present, evidence suggests that these subjects could benefit from starting or continuing appropriate immunomodulatory treatment. Nevertheless, due to the wide clinical variation in this group of patients, it is essential to tailor and personalize treatment choices. 

## 7. Conclusions

This comprehensive review summarized currently available data on the clinical and therapeutic aspects of people with LOMS. We also highlighted the main unmet needs in this field. The demographic age shift in the MS population necessitates a heightened awareness of comorbidities and treatment implications. In this scenario, the poorer prognosis in LOMS suggested by several studies is likely the consequence of multiple factors both related to the disease course and the advanced age (e.g., multiple morbidities, frequent clinical complications, difficulties in treatment). 

Advanced age impacts treatment decisions in MS, underscoring the necessity for tailored therapeutic strategies in LOMS. Notably, there exists a disparity in age between individuals with MS enrolled in regulatory drug trials and the aging MS population encountered in real-world settings. Many trials indicate a notable decrease in the anti-inflammatory efficacy of treatments among patients over the age of 40. Moreover, advanced age correlates with an elevated risk of experiencing adverse events, including serious infections. The presence of age-related comorbidities further complicates the risk–benefit assessment, occasionally leading to treatment discontinuation among patients. More studies are needed for a better understanding of the benefits of DMTs in LOMS, both in clinical trials and in the real-world setting.

## Figures and Tables

**Table 1:** Differentiating characteristics of late-onset multiple sclerosis (LOMS) and cerebral small vessel disease.

	LOMS	White Matter Hyperintensities	Lacunar Infarcts
**Age of presentation (years old)**	>50	>55	>55
**Shape**	Ovoid	Punctate, focal, and/or confluent	Round or ovoid
**Size**	>3 mm	Usually 3–12 mm	3–15 mm
**Sites**	Periventricular, cortical or juxtacortical, and infratentorial	Periventricular, adjacent to the lateral ventricular wall, or in the deep white matter	Basal ganglia, thalamus, subcortical white matter, and pons
**Clinic**	Often symptomatic	Silent	Silent or symptomatic
**MRI findings**			
T2/FLAIR	Hyperintense	Hyperintense	Hyperintense
Contrast enhancement	Variable	Absent	Variable

FLAIR, fluid-attenuated inversion recovery; LOMS, late-onset multiple sclerosis; MRI, magnetic resonance imaging.

**Table 2:** Differentiating magnetic resonance imaging (MRI) and laboratory features of myelopathy associated with late-onset multiple sclerosis (LOMS) and cervical spondylotic myelopathy.

	LOMS	Cervical Spondylotic Myelopathy
**Age of presentation (years old)**	>50	>50
**Level in cervical spine**	Upper part	Lower part
**Size**	Larger	Smaller
**Lesion border definition**	Well-defined	Ill-defined
**Thickness of the cord**	Normal	Decreased
**Edema**	Less frequent	More frequent
**Gadolinium enhancement**	In acute phases	Pancake-like or persistent enhancement
**CSF analysis**		
Oligoclonal bands	Common (less than AOMS)	Absent
Elevation of proteins	Mild, common	Mild/moderate, common
Pleocytosis	Mild, common	Rare

AOMS, adult-onset multiple sclerosis; CSF, cerebrospinal fluid; LOMS, late-onset multiple sclerosis.

## Data Availability

Not applicable.
